# Synthetic CT generation from CBCT using deep learning for adaptive radiotherapy in prostate cancer

**DOI:** 10.3389/fradi.2025.1680803

**Published:** 2025-11-13

**Authors:** Mustafa Çağlar, Kerime Selin Ertaş, Mehmet Sıddık Cebe, Ilkay Kara, Navid Kheradmand, Evrim Metcalfe

**Affiliations:** 1 Department of Health Physics, Graduate School of Health Sciences, İstanbul Medipol University, İstanbul, Türkiye; 2 Department of Radiation Oncology, Medipol Bahçelievler Hospital, İstanbul, Türkiye; 3 Program of Radiotherapy, Vocational School, İstanbul Medipol University, İstanbul, Türkiye; 4 Program of Radiotherapy, Vocational School and Health Services, İstanbul Medipol University, İstanbul, Türkiye; 5 Department of Radiation Oncology, School of Medicine, İstanbul Medipol University, İstanbul, Türkiye

**Keywords:** synthetic CT, CBCT, deep learning, image-guided radiotherapy (IGRT), adaptive radiotherapy

## Abstract

**Objective:**

In this study, the accuracy of deep learning-based models developed for synthetic CT (sCT) generation from conventional Cone Beam Computed Tomography (CBCT) images of prostate cancer patients was evaluated. The clinical applicability of these sCTs in treatment planning and their potential to support adaptive radiotherapy decision-making were also investigated.

**Methods:**

A total of 50 CBCT-CT mappings were obtained for each of 10 retrospectively selected prostate cancer patients, including one planning CT (pCT) and five CBCT scans taken on different days during the treatment process. All images were preprocessed, anatomically matched and used as input to the U-Net and ResU-Net models trained with PyTorch after z-score normalisation. The sCT outputs obtained from model outputs were quantitatively compared with the pCT with metrics such as SSIM, PSNR, MAE, and HU difference distribution.

**Results:**

Both models produced sCT images with higher similarity to pCT compared to CBCT images. The mean SSIM value was 0.763 ± 0.040 for CBCT-CT matches, 0.840 ± 0.026 with U-Net and 0.851 ± 0.026 with ResU-Net, with a significant increase in both models (*p* < 0.05). PSNR values were 21.55 ± 1.38 dB for CBCT, 24.74 ± 1.83 dB for U-Net, and 25.24 ± 1.61 dB for ResU-Net. ResU-Net provided a statistically significant higher PSNR value compared to U-Net (*p* < 0.05). In terms of MAE, while the mean error in CBCT-CT matches was 75.2 ± 18.7 HU, the U-Net model reduced this value to 65.3 ± 14.8 HU and ResU-Net to 61.8 ± 13.7 HU (*p* < 0.05).

**Conclusion:**

Deep learning models trained with simple architectures such as U-Net and ResU-Net provide effective and feasible solutions for the generation of clinically relevant sCT from CBCT images, supporting accurate dose calculation and facilitating adaptive radiotherapy workflows in prostate cancer management.

## 1 Introduction

Radiotherapy is a major component of cancer treatment and is applied in more than 50% of patients, either alone or in combination with surgery and systemic therapies. It aims to deliver radiation to the tumor site with high precision while minimizing damage to healthy tissues. Achieving this precision is critical for increasing the tumor control probability while reducing the normal tissue complication probability. Maintaining this balance in radiotherapy depends on several factors. Accurate delineation of target volumes and OARs is the primary factor in achieving effective and safe radiotherapy. Traditionally, computed tomography (CT) simulation has been considered the gold standard for this purpose (1). Another important factor is the accuracy of the patient's spatial position at the time of treatment. This accuracy is currently achieved by various Image Guided Radiotherapy (IGRT) methods. While kV portal imaging offers two-dimensional matching with digital reconstructed radiography (DRR) images obtained from CT, cone beam computed tomography (CBCT) provides volumetric imaging, enabling the monitoring of anatomical changes and treatment adaptation (2).

Soft tissue-based IGRT has replaced two-dimensional (2D) correction methods (3, 4). Among these online position correction methods, CBCT is the fastest and most accessible for conventional C-Arm and O-Ring linac systems (5). Thus, CBCT has become an indispensable component of patient positioning and daily treatment verification processes in radiotherapy clinics (1). The main advantage of CBCT compared to conventional CT is that it can be obtained on the treatment table and thus can respond immediately to anatomical changes. In this context, CBCT also forms the basis of adaptive radiotherapy (ART) applications (6). However, CBCT images are characterized by serious artifacts, low contrast resolution and most importantly inaccurate HU (Hounsfield Unit) values compared to conventional CT due to their low dose acquisition and differences in imaging geometries (7). These limitations seriously limit the use of CBCT in direct dose calculations and intensity-based segmentation/planning algorithms. In particular, failure to provide HU accuracy causes errors in dose distribution in photon/electron planning, while in sensitive modalities such as proton, even a few HU deviations can lead to deviations with intervals of a few millimeters (8, 9).

In order to improve the CBCT image quality, many methods have been proposed in the past (10–12). Among the hardware solutions, anti-scatter grid placement, ray blocking patterns or lead cages are noteworthy (13). Software solutions include methods such as iterative reconstruction (IR), ray tracing, model-based corrections and Monte Carlo (MC)-based simulations. Although MC-based approaches in particular provide successful results in terms of HU accuracy, they have not been put into clinical practice due to their high computational load (14).

The sCT generation has been proposed as an effective solution to resolve these challenges (10). This process involves the generation of CT-like images using various techniques such as bulk density assignment, atlas-based methods, hybrid methods, and deep learning-based approaches. Among these techniques, deep learning-based methods have proven to be the most the most promising approach (10, 15–18). In particular, direct sCT generation from CBCT images has become possible by using convolutional neural networks (CNN). Thus, the strategies used for CT generation from MRI have begun to be used in CBCT transformation as well as in low-dose CT enhancements (17, 19). Ryu et al. took these approaches a step further and proposed a new architecture called COMPUNet, which includes both multi-planar and 2.5D data approaches to improve CBCT quality. This model enhances learning efficiency, even when data availability is limited, and remains robust to slight spatial misalignments between input and output images. In addition, a contextual loss term is integrated in addition to the traditional L1 loss function. Thus, artifacts are attenuated while preserving details, and clinically significant improvements are achieved (20).

Zhang et al. presented one of the important studies in this field, generating images with CT-like appearance from pelvic CBCT images using a model based on the 2.5D Pix2pix GAN architecture. The model was strengthened with additional learning criteria such as feature matching and perceptual loss, and compared with different architectures (U-Net, CycleGAN). The results demonstrated that the MAE value was reduced to around 8 HU with the 2.5D GAN architecture, and the PSNR value reached 24 (21). Moreover, Kida et al. studied sCT generation from pelvic region CBCT with U-Net and reduced the MAE value from 92 to 31 HU (22). Zhu et al. introduced a residual block–based three-dimensional (3D) U-Net architecture combined with Dice–Focal loss to facilitate efficient, fully automated segmentation of head and neck anatomy (23). Liu et al. implemented a deep attention-based CycleGAN model in their study and achieved a reduction in MAE from 81 HU to 57 HU for the abdominal region (24).

Although GAN-based models can achieve high accuracy, they generally require larger datasets, longer training times, and greater computational resources, which can limit their clinical feasibility (15, 24, 25). Beyond CNN-based methods, diffusion models have been explored for CBCT-to-CT synthesis, reporting improved artifact suppression and high-frequency detail preservation (26–28). In parallel, transformer-based architectures, including Swin variants and hybrid CNN–transformer models, have been investigated for CBCT correction and sCT generation, leveraging long-range dependencies to enhance global consistency (29–31). These advanced approaches demonstrate strong performance but typically demand substantial data and computational resources, which may hinder their translation in resource-constrained clinical settings.

In this study, we focus on lightweight, relatively simple and resource-efficient CNN architectures, specifically U-Net and ResU-Net, as practical alternatives for prostate CBCT-to-sCT generation. These models are relatively simple, stable, and reproducible, making them more suitable for limited-data environments. Our contribution lies in demonstrating feasibility in a pilot study with multi-temporal CBCT data, where “multi-temporal” refers to multiple CBCT acquisitions obtained from the same patient across different treatment fractions (different days), from real patient treatments, emphasizing the clinical practicality of lightweight models under resource constraints. With this approach, we aim to show that deep learning models built on simpler architectures can still provide clinically relevant results and form a foundation for future integration into adaptive radiotherapy workflows.

## 2 Methods

### 2.1 Patient selection and imaging protocol

In this retrospective study, 10 patients diagnosed with prostate adenocarcinoma were randomly selected from the institutional radiotherapy archive and treated at the Radiation Oncology Department of Bahçelievler Medipol Hospital between 2019 and 2024. For each patient, one conventional planning CT scan and five separate CBCT images acquired on different days during the treatment course were included in the analysis. A total of 50 paired CBCT-CT image sets were generated. All image data were acquired in anonymized DICOM format and analyzed in accordance with the exemption granted by the local ethics committee (Non-Interventional Clinical Research Ethics Committee, Verification Code: 365043D5X6).

Planning CT images were acquired using a Siemens Biograph mCT 40 (Siemens Healthineers, Erlangen, Germany) scanner with a tube voltage of 120 kVp and a dose parameter of 132 mAs, resulting in voxel dimensions of 1.5 × 1.5 × 2 mm³. CBCT images were acquired using a Halcyon linear accelerator (Varian Medical Systems, Palo Alto, CA, USA) with a voxel size of 0.96 × 0.96 × 1.25 mm³ and a matrix size of 512 × 512 × 120. All images were registered for each patient, and CBCT scans were selected from different treatment fractions to ensure optimal anatomical consistency with the corresponding pCT.

### 2.2 Image preprocessing and slice matching

CBCT and CT datasets were imported and processed from DICOM format using the *SimpleITK* library within a Python 3.9 environment. To ensure spatial consistency, CBCT volumes were resampled to match the resolution of the corresponding planning CT scans, standardizing voxel dimensions across all datasets. A linear interpolation method was applied during the resampling process.

Each CBCT dataset was rigidly registered to the corresponding planning CT scan. Slice-wise alignment was then performed, and slices lying outside the overlapping anatomical region between CBCT and CT were excluded. In addition, slice correspondence was visually verified by a radiation oncologist and a medical physicist to ensure that key pelvic structures (e.g., prostate, rectum, bladder, femoral heads) were adequately represented. Slices showing severe mismatch due to differences in bladder/rectum filling or patient positioning were also discarded. After this process, a total of 1,890 slice pairs were retained, of which 1,323 were used for training, 283 for validation, and 284 for testing at the patient level.

This step was intended to isolate differences in image quality while minimizing the impact of anatomical variability. The alignment process was conducted individually for each patient, and only overlapping anatomical regions were used for model training. All images were normalized based on HU values using z-score normalization (mean = 0, std = 1) to standardize the input for model training. The normalized datasets were then converted to*.npy* format and used as input for the training process.

### 2.3 Deep learning architectures

This study employed two distinct fully convolutional neural network architectures for image translation. Each convolutional block consisted of two 3 × 3 convolutions with batch normalization and ReLU activation. In ResU-Net, identity skip connections were added to each block to stabilize training. Residual blocks enhanced feature extraction without a significant increase in parameters.

#### 2.3.1 U-Net

The U-Net architecture adopts the classical encoder–decoder design originally introduced by Ronneberger et al. 2D CBCT slice (256 × 256) is progressively downsampled in the encoder path through four convolutional blocks (channels: 32 →64 → 128 → 256 → 512), each block containing 3 × 3 convolutions, batch normalization, ReLU activation, and max pooling (32). The bottleneck layer consisted of 1,024 filters with a dropout rate of 0.5. The decoder path symmetrically upsamples the feature maps (channels: 512 → 256 → 128 → 64 →32) using 2 × 2 up-convolutions, concatenation with encoder features via skip connections, and subsequent convolutional blocks. This design preserves both global and local contextual information during reconstruction of the synthetic CT output.

#### 2.3.2 ResU-Net

ResU-Net incorporates residual connections (identity mappings) into each convolutional block of the original U-Net architecture. The encoder follows the same progression of channels (32 →64 → 128 → 256 → 512), with each block implemented as a residual block to enhance gradient flow and training stability. The bottleneck consisted of 1,024 filters with dropout (0.5) and residual blocks. The decoder similarly performs upsampling (channels: 512 → 256 → 128 → 64→32) using 2 × 2 up-convolutions followed by residual blocks. This integration mitigates the vanishing gradient problem and allows for deeper feature extraction without a significant increase in model complexity.

All model architectures were implemented from scratch in Python using the PyTorch 1.13.1 framework. Model training was conducted locally on a dedicated workstation featuring an NVIDIA GeForce GTX 1660 GPU (NVIDIA Corporation, Santa Clara, CA) with 6 GB of memory.

### 2.4 Training protocol

The model was trained on a slice-wise basis. One-to-one correspondence between CBCT and CT slices was ensured for each mapping. Both models were trained using a combined L1 and mean squared error (MSE) loss function, chosen to balance structural preservation with HU fidelity. Models were trained for 200 epochs with a batch size of 1 using the Adam optimizer (learning rate 1 × 10^−4^, β1 = 0.9, β2 = 0.999). Early stopping with a patience of 20 epochs was employed to prevent overfitting. Data augmentation included random horizontal/vertical flips and rotations within ±10°. Data were normalized to [0,1], and all images were resampled to a uniform voxel spacing of 1 × 1 × 1 mm³ prior to training. Training and validation data were split using slice-level randomization, with 80% allocated for training and 20% for validation. The schematic diagrams of the U-Net and ResU-Net architectures are provided in Supplementary Figures S1a,b, respectively.

### 2.5 Performance evaluation metrics

The outputs of the model, sCT images, were compared with the reference pCT and evaluated using several quantitative metrics. These included Structural Similarity Index (SSIM), measuring structural resemblance; Peak Signal-to-Noise Ratio (PSNR), Mean Absolute Error (MAE), representing the average HU difference; and the histogram of direct HU differences. Statistical analyses were performed using two-tailed Student's t-test, with *p* < 0.05 considered statistically significant. The performance of U-Net and ResU-Net models was analyzed individually for each metric, and improvements over the original CBCT images were quantitatively demonstrated.

## 3 Results

In this study, the effectiveness of U-Net and ResU-Net architectures was evaluated for generating sCT images from CBCT scans, aiming to achieve image quality comparable to pCT. Following the training and testing of the models on 50 CBCT–CT image pairs obtained from 10 patients, the resulting sCT images were quantitatively compared to the corresponding reference CT using a set of established image quality metrics.

### 3.1 Quantitative evaluation

Both models generated sCT outputs that demonstrated improved similarity to the reference CT images compared to the original CBCT scans. Comparative analyses were performed for both CBCT–CT and sCT–pCT pairs produced using the U-Net and ResU-Net architectures. Evaluation metrics included SSIM, PSNR, MAE, and average HU difference. The mean and standard deviation values for each metric, along with statistical comparisons for significance, are summarized in Table 1.

**Table 1:** Mean ± standard deviation values of all evaluation metrics and statistical significance comparisons for CBCT–CT, U-Net sCT–CT, and resU-Net sCT–CT matches (*n* = 50 matched pairs; significance level set at *p* < 0.05).

Metric	CBCT–CT	U-Net sCT–CT	ResU-Net sCT–CT	*p*-value
SSIM	0.763 ± 0.040	0.840 ± 0.026	0.851 ± 0.026	a, b
PSNR (dB)	21.55 ± 1.38	24.74 ± 1.83	25.24 ± 1.61	a, b, c
MAE (HU)	75.2 ± 18.7	65.3 ± 14.8	61.8 ± 13.7	a, b, c

*p* < 0.05 was considered statistically significant. a Significant difference between CBCT–CT and U-Net sCT–CT. b Significant difference between CBCT–CT and ResU-Net sCT–CT. c Significant difference between U-Net sCT–CT and ResU-Net sCT–CT.

**Figure 1:**
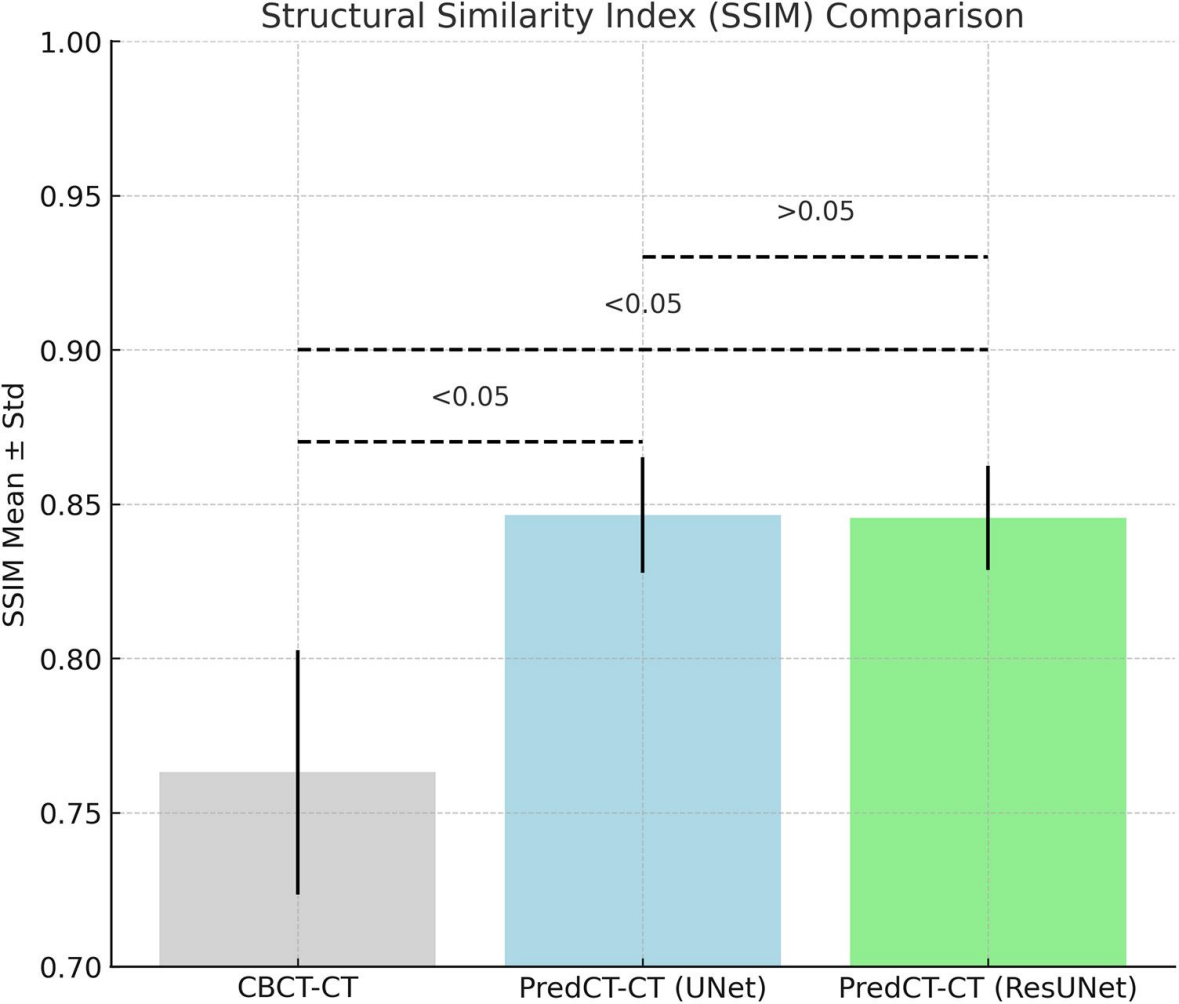
Comparison of SSIM values for CBCT–pCT, U-Net-based sCT–pCT, and resU-Net-based sCT–pCT pairs, presented as mean ± standard deviation. Statistically significant differences between groups (*p* < 0.05) are indicated on the plot.

According to Figure 1, both models significantly improved SSIM scores compared to the CBCT images (*p* < 0.05). While the mean SSIM was 0.763 ± 0.040 for CBCT–CT pairs, it increased to 0.840 ± 0.026 for U-Net sCT–CT and 0.851 ± 0.026 for ResU-Net sCT–CT. Statistical analysis confirmed that both U-Net and ResU-Net provided significant enhancements over CBCT; however, the difference between U-Net and ResU-Net was not statistically significant (*p* > 0.05).

**Figure 2:**
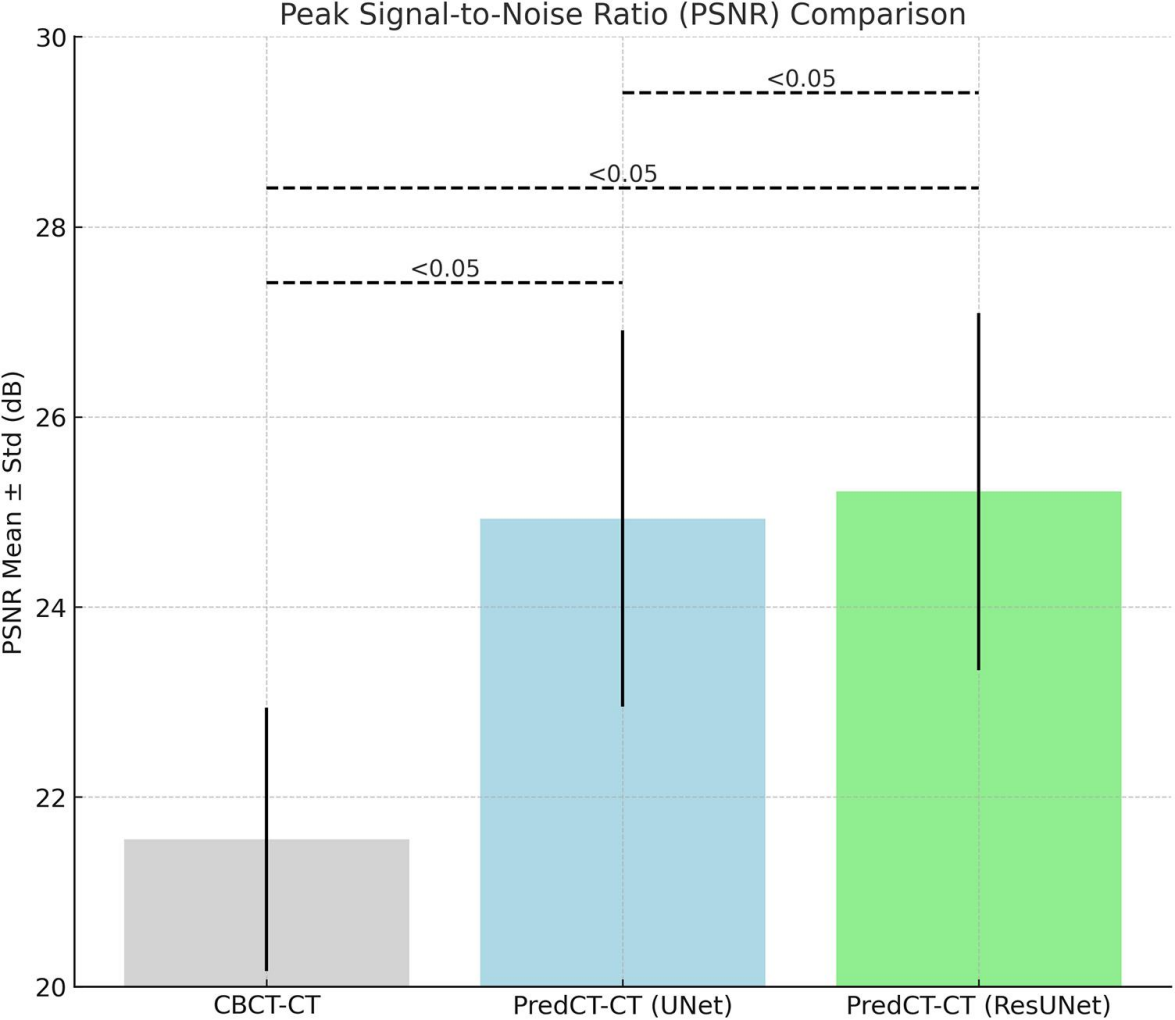
Comparison of PSNR (peak signal-to-noise ratio) values for CBCT–CT, U-Net-based sCT–CT, and resU-Net-based sCT–CT pairs, presented as mean ± standard deviation.

As shown in Figure 2, the mean PSNR for CBCT–CT pairs was 21.55 ± 1.38 dB, while U-Net sCT–CT and ResU-Net sCT–CT yielded 24.74 ± 1.83 dB and 25.24 ± 1.61 dB, respectively. Both models demonstrated significantly higher PSNR values compared to CBCT (*p* < 0.05). Moreover, the ResU-Net model significantly outperformed U-Net in terms of PSNR (*p* < 0.05).

**Figure 3:**
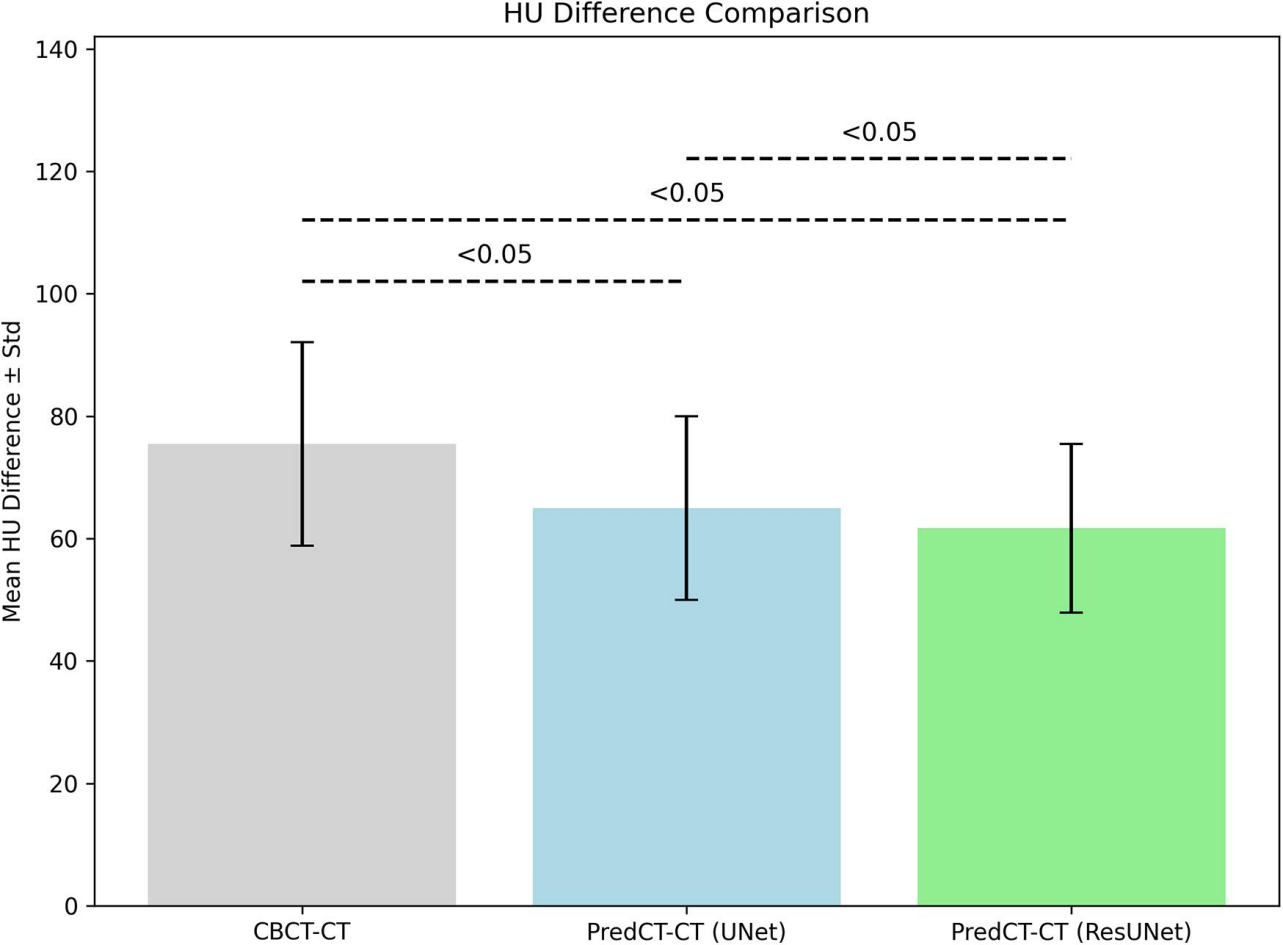
Comparison of the mean HU differences for CBCT–CT, U-Net sCT–pCT, and resU-Net sCT–pCT pairings.

**Figure 4:**
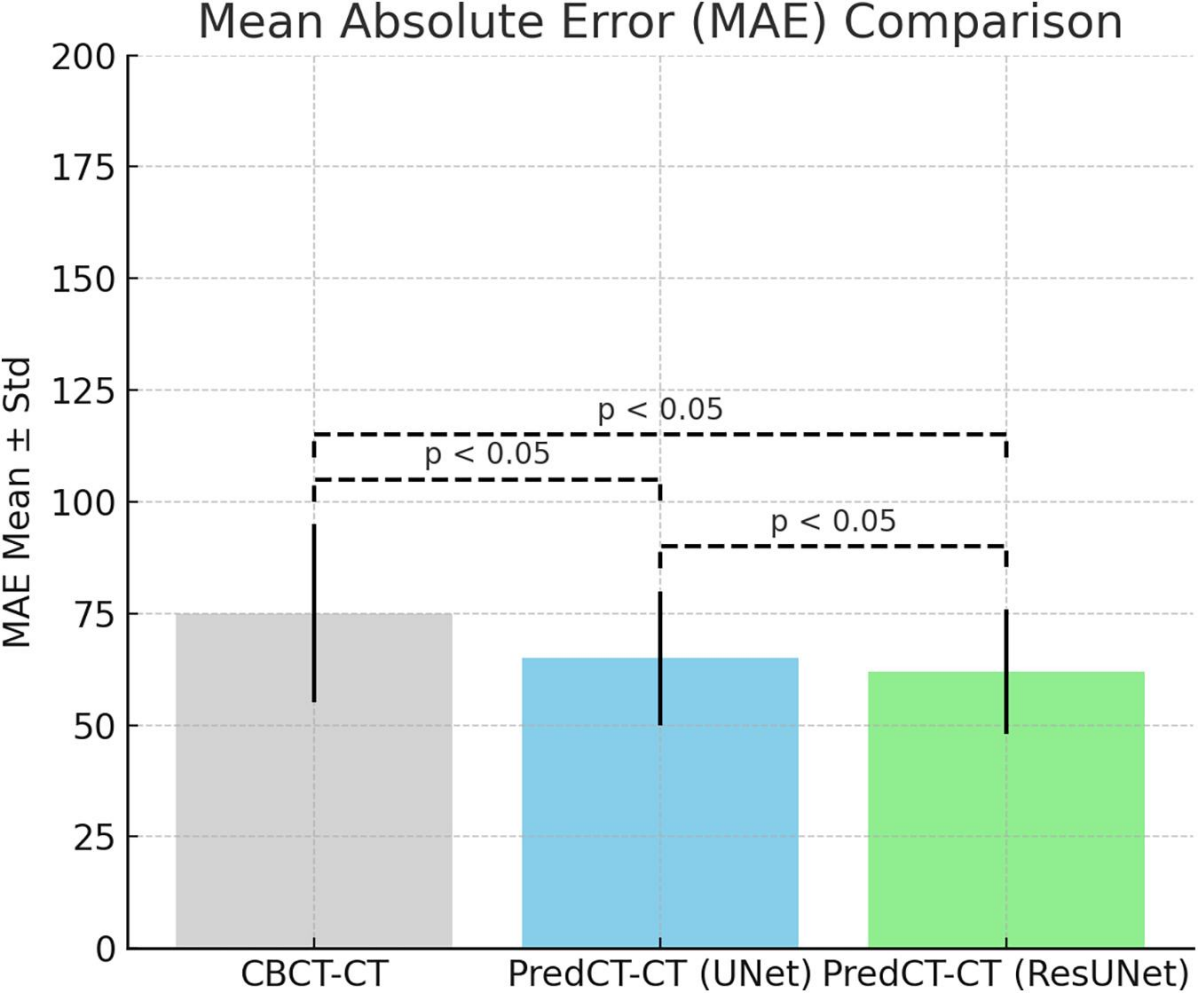
Statistical comparison of the mean absolute error (MAE) values for CBCT, U-Net, and resU-Net.

The mean HU difference for CBCT–CT pairs was 75.2 ± 18.7 HU. This was reduced to 65.3 ± 14.8 HU with U-Net and further to 61.8 ± 13.7 HU using ResU-Net (Figure 3). Both models showed significant improvements in HU accuracy over CBCT (*p* < 0.05), while ResU-Net also demonstrated a statistically significant advantage over U-Net (*p* < 0.05). Likewise, the MAE was 74.4 ± 36.8 HU on average in CBCT–CT, was reduced to 64.8 ± 29.6 HU with U-Net, and further decreased to 62.1 ± 30.0 HU with ResU-Net (Figure 4).

Based on these findings, although both models effectively improved the similarity of CBCT images to the pCT, the ResU-Net architecture achieved superior performance particularly in terms of structural similarity and reduction of reconstruction errors.

### 3.2 Qualitative evaluation

**Figure 5:**
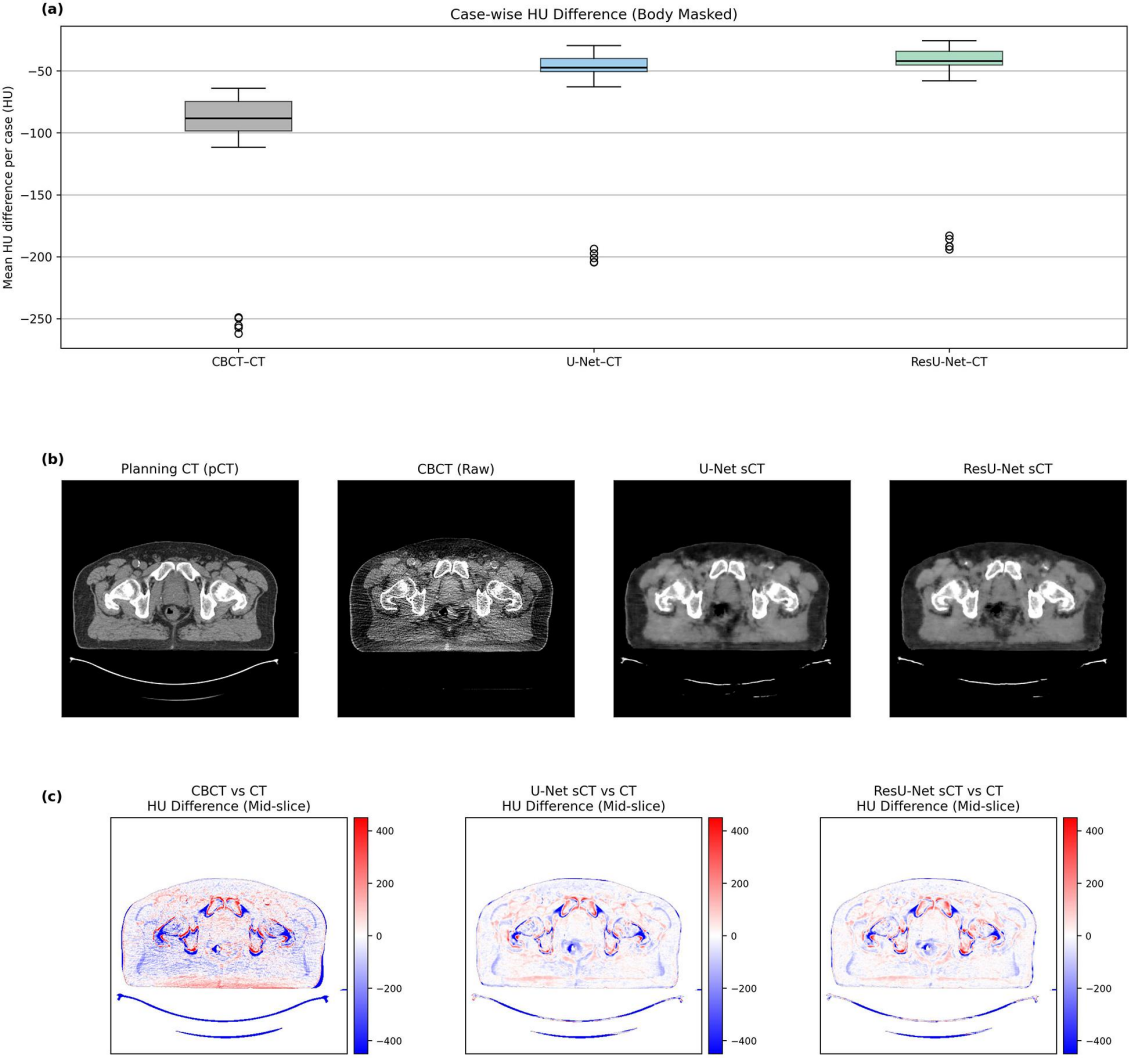
**(a)** Case-wise boxplots of mean HU differences for CBCT–pCT, U-Net sCT–pCT, and resU-Net sCT–pCT pairs (body-masked). **(b)** The mid-slice of CBCT10.1 (patient 10, fraction 1), including the planning CT, the raw CBCT, and the synthetic CT images generated by U-Net and ResU-Net, enabling a direct qualitative comparison of anatomical structures and visual fidelity across modalities. **(c)** Corresponding HU difference maps of CBCT, U-Net sCT, and ResU-Net sCT relative to the reference planning CT in the same mid-slice.

However, evaluating model performance requires not only analyzing mean HU differences but also examining their distributional characteristics. For this purpose, HU difference boxplots were generated, summarizing the central tendency, range, and outliers for all test cases (Figure 5a). The results demonstrate that raw CBCT images exhibit large deviations and outliers, particularly toward the negative HU range. While the U-Net model was able to reduce the spread of HU differences, the ResU-Net architecture produced even tighter distributions, reflecting improved robustness and consistency across patients.

Figure 5b displays the mid-slice of CBCT10.1 (patient 10, fraction 1) the planning CT, the raw CBCT, and the synthetic CT images generated by U-Net and ResU-Net, enabling a direct qualitative comparison of anatomical structures and visual fidelity across modalities. Figure 5c presents the mid-slice difference maps that visually reflect the anatomical alignment indicated by the quantitative analyses. In the CBCT map, pronounced deviations are visible, particularly at bone-to-soft tissue boundaries. While the U-Net model managed to reduce these discrepancies to a certain extent, ResU-Net achieved a more even correction of HU values and produced outputs that more closely resembled the reference CT. When considered alongside the numerical results, these visual observations reinforce the overall impression that ResU-Net provides a more consistent and dependable representation of HU information.

## 4 Discussion

In this study, the performance of the deep learning based model developed to generate sCT from conventional CBCT images was evaluated with quantitative metrics such as SSIM, PSNR, MAE, and HU. The results show that the closeness of the generated sCT images to the original CT images is significantly increased compared to the raw CBCT images.

The SSIM analysis demonstrated that both U-Net and ResU-Net architectures more effectively preserved structural information compared to conventional CBCT. The mean SSIM value for CBCT–CT alignment was 0.76, whereas it improved to 0.84 with U-Net and 0.85 with ResU-Net. These improvements were statistically significant when compared to CBCT. However, the slight advantage of ResU-Net over U-Net did not reach statistical significance. Although the SSIM values reported here are lower than the 0.93–0.98 range achieved by GAN-based models as described by Maspero et al., the results remain clinically acceptable given the model's simplicity and the limited dataset used (33).

The PSNR metric was employed to assess the signal fidelity of the generated sCT images. The average PSNR, calculated as 21.55 dB for CBCT–CT comparisons, increased to 24.74 dB with U-Net and 25.24 dB with ResU-Net. Both models yielded statistically significant improvements compared to CBCT, and ResU-Net demonstrated a notably higher PSNR than U-Net (*p* < 0.05). This result indicates that ResU-Net enhances sCT quality by more effectively preserving anatomical structures. The obtained values are consistent with the acceptable PSNR range for sCT quality, reported in the literature to be between 25 and 28 dB (17, 34).

A similar pattern was observed in the MAE results. The average MAE for CBCT–CT alignment was measured at 75.2 HU, which decreased to 64.1 HU with U-Net and 61.8 HU with ResU-Net. Both deep learning models achieved statistically significant reductions in error compared to CBCT (*p* < 0.05). Furthermore, ResU-Net demonstrated a modest yet statistically significant advantage over U-Net, indicating that residual connections enhance the suppression of HU-related discrepancies. In previous research, Wongtrakool et al. reported reducing the mean MAE to approximately 58 HU using a GAN-based approach in the head and neck region (17). While the MAE values obtained in our study are slightly higher than those reported in some previous study and commercial implementations, they nevertheless highlight the feasibility of lightweight CNN architectures in improving CBCT image quality. These findings should be interpreted as proof-of-concept results, with further optimization and larger datasets required to establish clinical acceptability. Despite its simpler architecture, the ResU-Net model employed in this study achieved comparably low MAE values, underscoring its potential to balance algorithmic efficiency with clinical relevance.

Figure 5 presents case-wise HU difference boxplots and mid-slice difference maps that visually support the quantitative findings. The boxplot analysis reveals that CBCT images exhibit a broad distribution with pronounced variability and outliers, whereas the U-Net model reduces this spread and the ResU-Net architecture further centralizes the HU values, indicating improved accuracy and consistency across patients. Similarly, mid-slice difference maps indicate prominent deviations at bone–soft tissue interfaces in CBCT images, whereas ResU-Net suppresses these discrepancies more uniformly, yielding an image that closely aligns with the pCT. Further inspection revealed that the outlier points in Figure 5A predominantly originated from high-density bony regions and adjacent interfaces, where inherent HU gradients and CBCT-specific artifacts such as scatter and beam hardening cause local intensity deviations. Despite these localized discrepancies, the synthetic CTs exhibited consistent HU improvement over raw CBCT, particularly at bone–soft tissue boundaries, as also evident in Figure 5C. When considered collectively, these findings suggest that the ResU-Net architecture provides more stable and reliable performance in terms of HU accuracy, both in pixel differences and mean absolute error.

Although simpler than GAN-based models, the U-Net and ResU-Net architectures implemented in this study achieved performance levels consistent with previous research and suitable for feasibility assessment in clinical imaging quality improvement. For instance, the cGAN model used by O'Connor et al. for MR-based sCT generation demonstrated high SSIM scores and low HU deviations, but required a more complex and data-intensive training process (10). In contrast, the ResU-Net model, enhanced with residual connections, produced comparable results in both HU accuracy and structural similarity while preserving a relatively simple design.

In this context, the approaches introduced in this study offer alternative model architectures that are effective under limited data conditions and hold strong potential for clinical integration. Given that only 10 patients (50 paired CBCT–CT volumes) were included, the findings should be interpreted as a pilot feasibility study rather than a robust validation. We also acknowledge that the feasibility of deep learning–based CBCT enhancement has been demonstrated in earlier studies; however, our contribution emphasizes the applicability of lightweight architectures in prostate cancer and multi-temporal CBCT settings, highlighting a practical pathway under resource-constrained clinical conditions.

Another notable limitation is that the study was conducted using 2D slice-based architecture. In addition, although both U-Net and ResU-Net significantly reduced the MAE compared to CBCT, the absolute values remained modest and slightly higher than those reported in some previous studies and commercial systems, indicating that further optimization is needed before clinical acceptability can be established. Future research should therefore explore more advanced approaches, including three-dimensional CNNs, and incorporate larger, multi-center datasets to enhance model generalizability. Moreover, comprehensive evaluations are needed, including integration into clinical workflows, assessment of dose calculation accuracy, auto-contouring performance, and DVH integrity. Therefore, the present results should be interpreted as a feasibility study at the imaging quality level only, while future work must establish true clinical utility for radiotherapy applications.

## 5 Conclusion

These findings suggest that deep learning-based transformation methods, even those employing relatively simple architectures, can be effectively applied in clinical settings with limited data availability. The results indicate that U-Net, and especially ResU-Net, enhance CBCT image quality and enable reliable sCT generation, providing proof-of-concept feasibility in a pilot setting. This study highlights the clinical practicality of lightweight CNN architectures for sCT generation in prostate radiotherapy. Nevertheless, further validation with larger multi-institutional datasets and advanced methods is warranted. In particular, comprehensive evaluations such as dosimetric accuracy, auto-contouring performance, and DVH integrity are required before true clinical integration can be established.

## Data Availability

The original contributions presented in the study are included in the article/Supplementary Material, further inquiries can be directed to the corresponding author.
