# Supplementary figures and images for: Synthetic CT generation from CBCT using deep learning for adaptive radiotherapy in prostate cancer
Source: Front Radiol. 2025 Nov 13;5:1680803. doi: 10.3389/fradi.2025.1680803 (PMC12657355; doi:10.3389/fradi.2025.1680803)

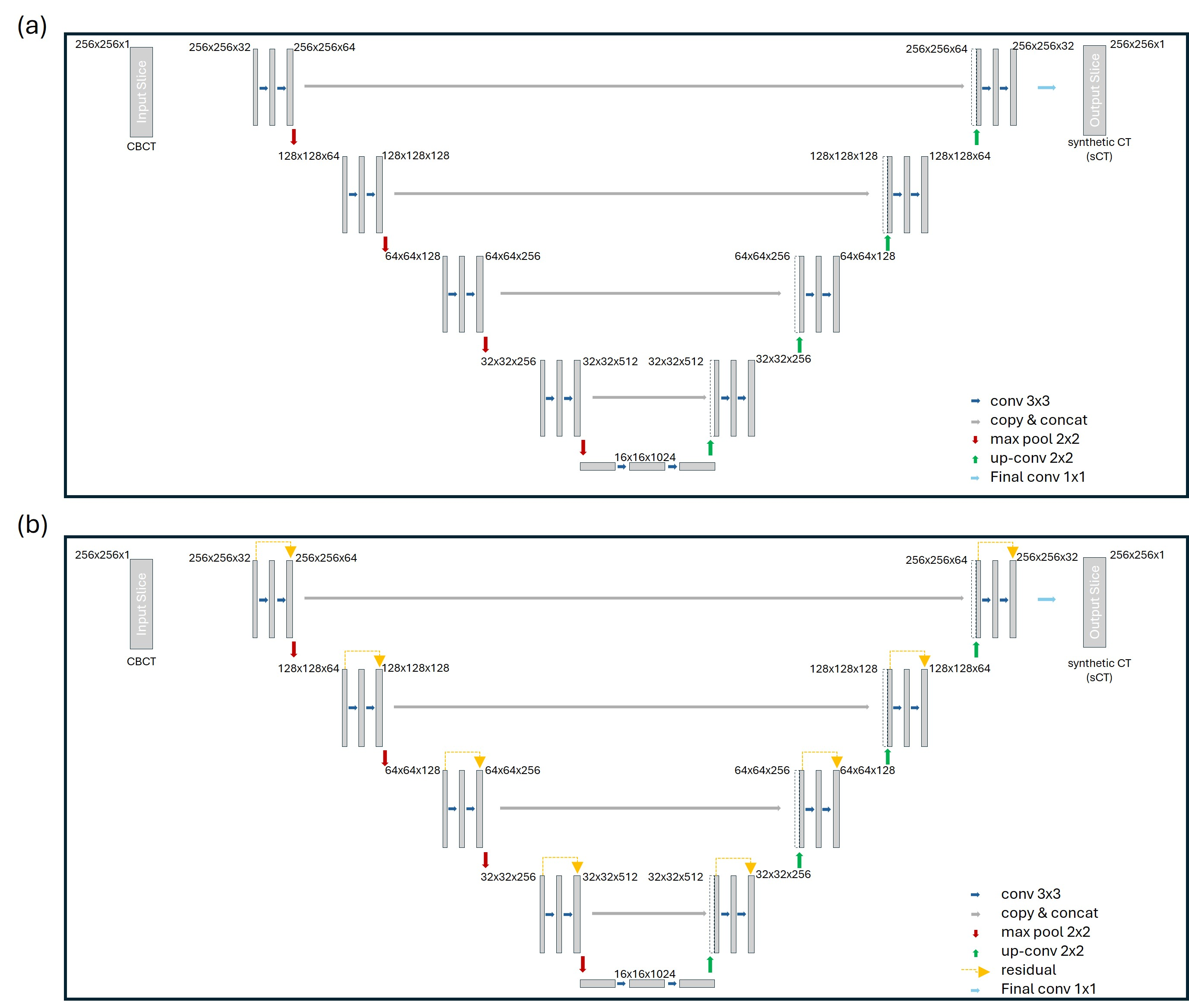

Supplement: Supplementary file 2 [file Image1.tiff]
